# Editorial: Cleft Lip and Palate Anomalies/Syndromes

**DOI:** 10.3389/fphys.2022.926269

**Published:** 2022-06-20

**Authors:** Mohammad Khursheed Alam, Mohammed Moniruzzaman, Kathiravan Purmal

**Affiliations:** ^1^ College of Dentistry, Jouf University, Sakaka, Saudi Arabia; ^2^ The Johns Hopkins Hospital, Johns Hopkins Medicine, Baltimore, MD, United States; ^3^ Columbia Asia Hospital, Kuala Lumpur, Malaysia

**Keywords:** cleft lip and palate (CLP), cleft lip and palate deformity, craniofacial abnormalities, genetics, dental anomalies, genome wide association, velopharyngeal (VP) insufficiency, quality of life

The topic “*Cleft Lip and Palate Anomalies/Syndromes*” is a Frontiers Research Topic aimed to provide an opportunity for researchers and clinicians from different perspectives and areas to publish recent advances in the understanding of Cleft lip and palate (CLP) anomalies/syndromes.

Cleft lip and palate (CLP) anomalies/syndromes are integrated in different subjects of medicine and dentistry. An abundance of research is ongoing in this field. Any deformities (anatomical or chromosomal) that are initiated during pregnancy, with their effects being detected at birth, are considered as congenital anomalies. Among them, CLP is one of the most common congenital anomalies in the head and neck region and only second to congenital heart disease in the whole body with varied prevalence in different civilizations and races. The World Health Organization (WHO) has recognized and included cleft deformities in their Global Burden of Disease initiative. It is estimated that the overall global prevalence of cleft deformities is one affected individual in every 600 new born babies. Though the exact etiology of CLP is controversial, it is believed that both genetic and environmental factors play an important role. It affects psychological development, causes aesthetic and functional problems such as feeding, speech, hearing, and dental functioning. In addition, it may also contribute in dentoskeletal abnormalities such as maxillary arch constriction (maxillary growth retardation), class III malocclusion, mid facial growth deficiency, congenitally missing and malformed teeth, and other orthodontic anomalies like crowding, rotation, malposition of teeth. Medical, surgical, dental, speech, and allied health in cleft lip and palate anomalies/syndromes are of utmost importance in the clinical, epidemiological and research field. The diverse and complex needs of patients with cleft lip and palate anomalies/syndromes, and the obligatory facilities of many varied professionals warrants a multidisciplinary approach for successful management.

In this topic, studies with genome-wide association study and genetics are presented by Ghazali et al. and Küchler et al.

Ghazali et al. aimed to identify the genetic aberration involved in both Nonsyndromic cleft lip and or without cleft palate (NSCL/P) and hypodontia pathogenesis. Original research with cross-sectional study using genome-wide study copy number variation-targeted CytoScan 750K array carried out on salivary samples are investigated. There were a significant gain and loss of both SKI and fragile histidine triad (FHIT) copy number in NSCL/P with hypodontia compared with the noncleft group were explored.

Küchler et al. explored the association between isolated tooth agenesis and genetic polymorphisms in genes that are crucial for craniofacial and tooth development. They reported, the TT genotype in rs3934908 (SMAD6) was associated with higher chance to present third molar agenesis. BMP2 was also associated in haplotype and diplotype analysis with tooth agenesis.

In the current topic, studies with human samples using model and cephalometric x-ray were also presented. Two studies investigated oral cleft patients. Haque et al. assessed of 3D digital models using GOSLON Yardstick Index. And explored confounding factors responsible for unfavourable treatment outcome in multi-population children with UCLP. Alam et al. investigated whether the craniofacial sagittal jaw relationship in patients with different type of non-syndromic cleft differed from non-cleft (NC) individuals by artificial intelligence (A.I.)-driven lateral cephalometric (Late. Ceph.) analysis. And the study advocates a decrease in sagittal development (SNA, ANB and Wits appraisal) in different types of cleft compared to NC individuals.

A systematic review from Chen et al. explored giving new insight into current Velopharyngeal Inadequacy-Related Quality of Life Assessment (VPI-related QOL) instrument development, validation, and applicability. In this review, understanding the development and characteristics of different QOL instruments, including their reliability, validity, aim, target, language, and resource, should be important before application in clinic or research.

Human samples were also used to explore the effect of bilateral mandible distraction osteogenesis about the nutrition status of infants with pierre-robin sequence by Jiyau et al. They reported, bilateral mandible distraction osteogenesis surgery has a positive effect on the nutrition status of children with pierre-robin sequence. This effect is mainly reflected by the improvements of the body physical indicators after surgery. And, Yang et al. conducted a preliminary study using computational fluid dynamic analysis, inspiration after posterior pharyngeal flap palatoplasty. Real-time computational fluid dynamics simulation was used to capture the airflow through the ports. Posterior pharyngeal flap palatoplasty is one of the most common-used surgical procedures to correct speech, especially for patients suffering from velopharyngeal insufficiency. They found that the airflow dynamics of the upper airway’s inspiration were dependent on the velopharyngeal structure. Although the airflow patterns were similar, the velocities between the one-port and two-port structures were different, which explained why patients after posterior pharyngeal flap palatoplasty breathed harder than before and suggested a one-port structure might be a better choice for secondary velopharyngeal insufficiency reconstruction based on the computational fluid dynamics analyses.

This Research Topic targets the acquisition and dissemination of knowledge regarding all aspects (including etiology, prevention, diagnosis, treatment and its outcome) of cleft lip and palate anomalies/syndromes to share knowledge from both the basic and clinical sciences. Briefly, this Research Topic provided an opportunity for researchers and clinicians from different perspectives and areas to discuss recent advances in the understanding of Cleft Lip and Palate Anomalies/Syndromes. It also will provide readers with new insights and different viewpoints to stimulate further investigations in this broad research field. This Research Topic also achieved its initial aim to have different areas contemplated, such as genome-wide association, genetic polymorphisms in genes involved in craniofacial development and isolated tooth agenesis, 3D digital model assessments, artificial-intelligence based craniofacial assessment, quality of life research, coming together in this article Research Topic.

